# Are Diabetic Patients at Increased Risk for Biochemical Recurrence After Radical Prostatectomy?

**DOI:** 10.7759/cureus.24717

**Published:** 2022-05-04

**Authors:** Houssem Ben Hadj Alouane, Mehdi Raboudi, Jasser Maatougui, Mohamed Dridi, Samir Ghozzi

**Affiliations:** 1 Department of Urology, Military Hospital of Tunis, Tunisia, TUN

**Keywords:** risk factor, outcome, biochemical recurrence, prostate cancer, diabetes mellitus

## Abstract

Introduction

Diabetic patients are at a lower risk for prostate cancer. However, the relationship between diabetes mellitus (DM) and biochemical recurrence (BCR) after radical prostatectomy (RP) is less clear. The goal of our study was to determine diabetes's value as a biochemical recurrence predictor.

Materials and methods

We conducted a retrospective analysis of 117 patients who had undergone open radical prostatectomy between 1999 and 2021 at our institution. Univariate and multivariate statistical analyses were used to identify factors associated with biochemical recurrence.

Results

On univariate analysis, factors associated with biochemical recurrence were diabetes (p=0.002), preoperative prostate-specific antigen (PSA) levels (p=0.022), positive digital rectal exam (p=0.035), number of positive biopsy cores (p<0.001), unfavorable intermediate risk group (p=0.014), peri-neural invasion (PNI) on RP specimen (p=0.043), tumor volume (p=0.011), and positive surgical margins (p<0.001). Multivariate analysis showed that factors independently associated with biochemical recurrence were diabetes (p=0.039; OR=2.788), number of positive cores (p=0.016; OR=4.124), and positive surgical margins (p=0.008; OR=3.876).

Conclusion

A history of diabetes mellitus should be taken into consideration when assessing patients’ risk of biochemical recurrence after radical prostatectomy. More research on a larger scale is needed to determine diabetes' value as a biochemical predictor.

## Introduction

The relationship between diabetes mellitus (DM) and neoplasms varies from one disease to another. It is known to be a risk factor for many cancers, such as colorectal [1,2], lung [3], and nasopharyngeal cancers [4]. At the same time, it seems to have an inverse relationship with prostate cancer [5]. However, when a patient already presents with prostate cancer, diabetes seems to be associated with increased cancer-specific mortality [6]. Biochemical recurrence (BCR) after radical prostatectomy (RP) for prostate cancer represents the primary oncological outcome and the first sign of clinical progression. It affects 27-53% of patients treated for localized prostate cancer [7], and it is associated with various adverse endpoints such as increased progression to metastasis and disease-specific mortality [8]. Diabetes is particularly prevalent among the elderly being treated for prostate cancer. Therefore, it is necessary to identify its ability to predict poor oncological outcomes. The goal of our study was to assess the relationship between diabetes and biochemical recurrence in patients treated with radical prostatectomy for localized prostate cancer.

## Materials and methods

Data collection and patient selection

After gaining local ethics committee approval, the surgical records of our institute were reviewed to identify patients who had undergone open radical prostatectomy between January 1999 and December 2021 for localized prostate cancer. Clinical characteristics and follow-up data were collected from the patients’ charts. Patients who were diagnosed with DM before surgery were considered to have a history of DM.

Patients were seen at three months post-RP, then semiannually. Laboratory measurement of prostate-specific antigen (PSA) was performed at each visit. The BCR was defined by the post-operative increase in PSA > 0.2 ng/ml, confirmed by two successive tests.

Of the 140 identified patients, we excluded those whose PSA did not decrease beyond the 0.2 ng/ml threshold (n = 15). We also excluded from the analysis patients who had undergone radiotherapy or hormonal therapy, which would interfere with oncological outcomes (n = 8).

Statistical analysis

After exclusion, 117 patients remained for statistical analysis. A comparison of the clinical characteristics between the diabetic and non-diabetic groups was made. A one-way ANOVA for continuous variables and a chi-square test for categorical variables were used. BCR-free survival curves were generated using the Kaplan-Meier method, and a log-rank test was applied for pairwise comparison of survival. A multivariable Cox regression model addressed the association of DM with BCR after RP. All p-values were two-sided, and statistical significance was defined as p < 0.05. Statistical analyses were performed using SPSS Statistics 26 (SPSS, IBM Corp, Armonk, NY, USA).

## Results

Descriptive analysis

The clinical and demographic characteristics of our patients are detailed in Table 1.

**Table 1:** Clinicopathologic characteristics of 117 patients who underwent radical prostatectomy DRE: digital rectal examination; PNI: peri-neural invasion; RP: radical prostatectomy

Variables	Value
Median age, years	64
Diabetes (n,%)	39 (33.3%)
Mean PSA	9.3
Lesion on DRE (n,%)	26(22,2%)
Median positive biopsy cores	3
Biopsy Gleason (n, %)	
6	68(58.1%)
7	46(39.4%)
≥8	3(2.5%)
Biopsy PNI (n,%)	17(14.5%)
NCCN risk score (n,%)	
Very low	6(5.1%)
Low	36(30.8%)
Favorable intermediate	34(29.1%)
Unfavorable intermediate	25(21.4%)
High risk	16(13.7%)
RP specimen Gleason (n,%)	
6	46(39.4%)
7	67(57.4%)
≥8	4(3.2%)
RP specimen PNI (n,%)	37(31.6%)
Clinical stage (n, %)	
T2a	36(30.8%)
T2b	7(6%)
T2c	61(52.1%)
T3a	12(10.3%)
T3b	1(0.9%)
Mean tumor volume	24%
Positive surgical margins (n,%)	29(24.8%)

There were a total of 39 diabetic patients (33.3%). On univariate analysis, there were no significant differences between the diabetic and the non-diabetic sub-groups except for the BCR rate, which was significantly higher in the DM group (p=0.002). Both groups presented with similar prognostic characteristics such as initial PSA (p=0.9), definitive Gleason score (p=0.7), extra-capsular extension (p=0.6), and positive surgical margins (p=0.1). The results of the comparison are detailed in Table 2.

**Table 2:** Comparison between the diabetic and non-diabetic subgroups PNI: peri-neural invasion; RP: radical prostatectomy

	Diabetic patients (n=39)	Non-diabetic patients (n=78)	P-value
Mean age, years	64.5	64.2	0.8
Mean PSA	9.8	9.7	0.9
Mean positive biopsy cores	3.87	3.07	0.051
RP Gleason score (n, %)			0.7
6	14 (35.9%)	32 (41%)	
7	23 (59%)	44 (56.4%)	
≥8	2 (5.1%)	2 (2.6%)	
RP extra-capsular extension (n, %)	4 (10.3%)	8 (10.2%)	0.6
RP PNI (n, %)	16 (41%)	21 (26.9%)	0.09
Positive surgical margins (n, %)	13 (33.3%)	16 (20.5%)	0.1
Biochemical recurrence (n, %)	15 (38.5%)	10 (12.8%)	0.002

Biochemical recurrence analysis

**Figure 1:**
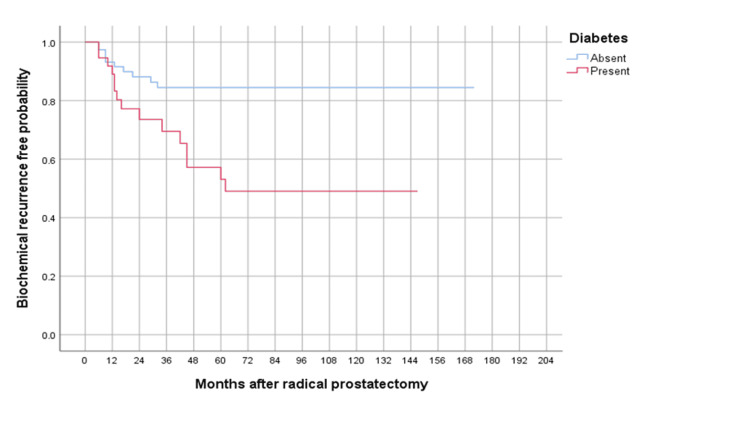
Kaplan–Meier curves depicting biochemical recurrence-free survival in 117 patients, according to diabetes status

The mean follow-up for our patients was 64.8 months. Of the 117 patients, 25 (21.4%) experienced BCR. The mean time-to-BCR was 22.1 months. Of the 39 diabetic patients, 38.5% experienced BCR, while the BCR rate in the non-diabetic group was 12.8%. After establishing Kaplan-Meier survival curves and subsequent log-rank testing, DM was found to be negatively associated with the BCR-free survival rate (p=0.002; Figure 1).

Other significant factors associated with BCR on univariate analysis included preoperative PSA levels (p=0.022), positive digital rectal exam (p=0.035), number of positive biopsy cores (p<0.001), unfavorable intermediate-risk group (p=0.014), perineural invasion on RP specimen (p=0.043), tumor volume (p=0.011), and positive surgical margins (p<0.001). The Cox-regression model was established using the variables found to be significantly associated with BCR. After multivariate analysis, factors independently associated with BCR were diabetes mellitus, number of positive cores, and positive surgical margins. Results from the univariate and multivariate analyses are detailed in Table 3.

**Table 3:** Univariate and multivariate Cox regression analyses predicting BCR after RP DRE: digital rectal examination; NCCN: National Comprehensive Cancer Network; RP: radical prostatectomy; PNI: peri-neural invasion

Variable	Univariate p-value	Multivariate analysis	
		OR (95% CI)	p-value
Diabetes	0.002	2.788 (1.055-7.366)	0.039
Suspect DRE	0.04	1.113 (0.347-3.574)	0.85
PSA ≥ 8 ng/ml	0.02	1.474 (0.489-4.442)	0.491
Positive biopsy cores ≥ 6	0.001	4.124 (1.307-13.016)	0.016
Biopsy PNI	0.98	-	-
Biopsy Gleason score	0.46	-	-
NCCN ≥ unfavorable intermediate	0.014	0.56 (0.166-1.888)	0.35
RP Gleason score	0.66	-	-
RP PNI	0.043	0.839 (0.296-2.377)	0.714
Clinical stage	0.58	-	-
Tumor volume ≥ 10%	0.011	2.970 (0,614-14.355)	0.176
Positive surgical margins	<0.001	3.876 (1.430-10.504)	0.008

## Discussion

In our study, the number of positive biopsy cores, positive surgical margins, and diabetes were independent predictors of BCR. While biopsy core invasion [9] and surgical margins [10] are relatively well-established BCR risk factors, the data surrounding diabetes is both scarce and divided. In a case-control study comparing 210 diabetic patients to 406 control cases, Patel et al. [11] showed that diabetes was independently associated with BCR (p=0.034; HR=1.55). The same results were found in two more recent studies [12,13].

This positive association comes in contrast to other authors. Through a cohort of 6722 patients, Chan et al. showed that the relation between diabetes and BCR was not significant (p=0.45) [14]. More recent studies also failed to establish a link between diabetes and BCR [15,16]. These contradictory findings suggest that diabetes could interact with PC cells on different levels.

Diabetes could have a potentially protective role against PC progression by reducing the activity of insulin-like growth factor 1 [17] and testosterone levels [18]. On the other hand, elevated glucose levels, hyperinsulinemia, and immunodeficiency represent some of the mechanisms by which diabetes could induce PC growth and BCR [19]. Diabetes is a complex disease featuring numerous pathological mechanisms. Studying its components seems necessary in order to shed light on the relationship with BCR.

The relationship between metformin usage and BCR has been studied as in vitro data suggested that metformin might induce apoptosis in prostate cancer cells [20]. Conflicting data also surround this hypothesis. Spratt et al. [21], through a retrospective study of 2901 patients, found that metformin is associated with improved BCR-free survival, metastasis-free disease, and decreased cancer-specific mortality. One meta-analysis provided further proof of the protective role of metformin as it was inversely associated with BCR-risk (p = 0.047; OR=0.79) [22]. Conversely, many other studies found the relationship between metformin use and BCR was not significant [11,15,23,24].

Another variable associated with diabetes is the HbA1C level, which seems to be associated with poor oncological outcomes [25]. Indeed, it is wrong to assume that a simple history of diabetes entails poor glycemic control. This notion was studied by Lee et al. [26] in a cohort of 746 patients where HbA1c levels were analyzed in relation to BCR. The results have shown that although a history of diabetes was not associated with BCR, high HBA1c levels (⩾6.5%) were associated with worse BCR-free survival on multivariate analysis (p=0.024; HR=1.135). The results were similar in an observational study of 1249 patients led by Galina et al. [27], where high HBA1c levels independently predicted BCR (p=0.02) as well as seminal vesicle invasion and lymph node involvement. These findings might explain the discrepancy between the previous results as glycemic control varies depending on geographical and socio-economic factors. Indeed, in the case of our country, glycemic levels within diabetic patients tend to be poorly regulated, which is consistent with our results.

Obesity seems to be another potential confounder, explaining the inconsistent link between diabetes and BCR. While failing to find a significant association between diabetes and BCR in his overall cohort, Jayachandran et al. [28] noted that diabetes was associated with a 2.5-fold increase in BCR risk in the obese subgroup (P=0.002). This claim is supported by many other studies reinforcing the relationship between obesity and BCR [13,29,30].

While the homogeneity between the groups and long follow-up period represent strengths in our study, our main limitation is the relatively small cohort which mirrors the general population of our country. Our results however constitute further evidence of diabetes’ value as a prognostic factor.

## Conclusions

In conclusion, our study seems to suggest that a history of diabetes, along with the number of positive biopsy cores and the surgical margin status, should be taken into consideration when assessing biochemical recurrence risk after radical prostatectomy. This could allow for a better selection of candidates for complementary treatment after RP. Diabetes prognostic value seems to be influenced by other covariates such as obesity and glycemic control. More research, both epidemiological and mechanistic, is needed to further clarify the relationship between diabetes and the risk of PC BCR.
